# Supplementary material for: Cessation of Nucleos(t)ide Analogue Therapy in Non-Cirrhotic Hepatitis B Patients with Prior Severe Acute Exacerbation
Source: J Clin Med. 2021 Oct 23;10(21):4883. doi: 10.3390/jcm10214883 (PMC8584579; doi:10.3390/jcm10214883)
Supplement: Supplementary file 1 [file jcm-10-04883-s001.zip › jcm-1416975-supplementary.pdf]

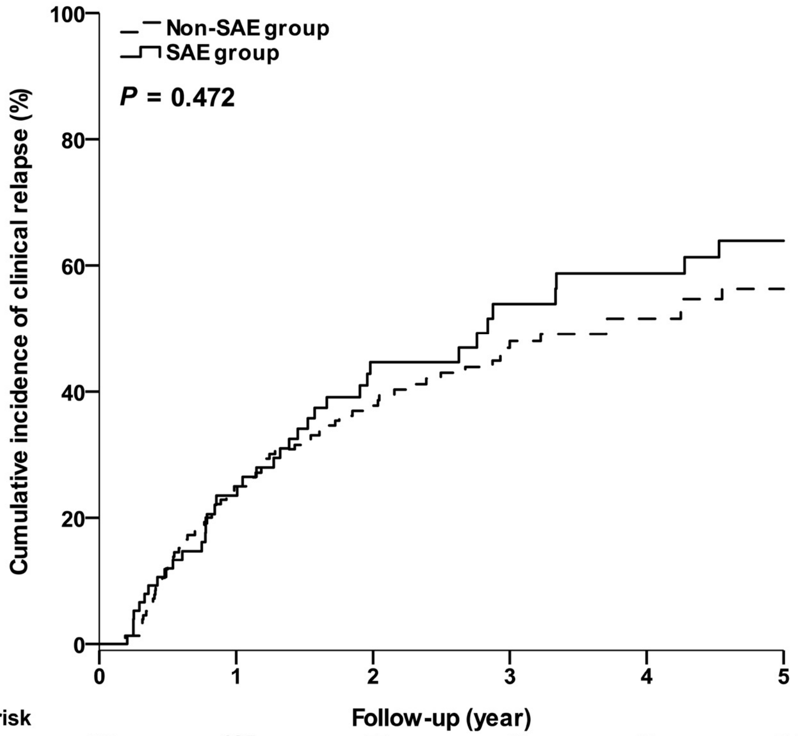

Number at risk						
Non-SA group	156	105	75	49	36	25
SAE group	78	52	30	20	17	12

Figure S1. The cumulative incidences of clinical relapse.

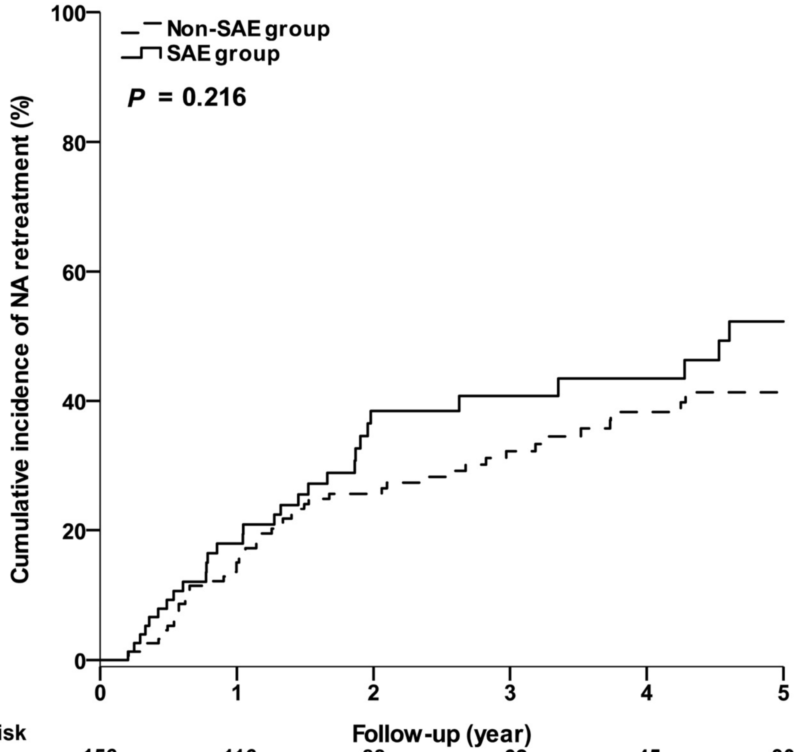

Number at risk						
Non-SAE group	156	116	88	62	45	30
SAE group	78	56	32	24	21	14

Figure S2. The cumulative incidences of NA retreatment.
